# Disease Transmission by Misfolded Prion-Protein Isoforms, Prion-Like Amyloids, Functional Amyloids and the *Central Dogma*

**DOI:** 10.3390/biology5010002

**Published:** 2016-01-04

**Authors:** Martin L. Daus

**Affiliations:** ZBS6-Proteomics and Spectroscopy, Robert Koch-Institute, Seestrasse 10, 13353 Berlin, Germany; dausm@rki.de; Tel.: +49-30-187-542-844; Fax: +49-30-187-542-664

**Keywords:** prion, *Central Dogma*, protein misfolding, amyloid, prion-like amyloids, functional amyloids

## Abstract

In 1982, the term “prions” (proteinaceous infectious particles) was coined to specify a new principle of infection. A misfolded isoform of a cellular protein has been described as the causative agent of a fatal neurodegenerative disease. At the beginning of prion research scientists assumed that the infectious agent causing transmissible spongiform encephalopathy (TSE) was a virus, but some unconventional properties of these pathogens were difficult to bring in line with the prevailing viral model. The discovery that prions (obviously devoid of any coding nucleic acid) can store and transmit information similarly to DNA was initially even denoted as being “heretical” but is nowadays mainly accepted by the scientific community. This review describes, from a historical point of view, how the “protein-only hypothesis” expands the *Central Dogma*. Definition of both, the prion principle and the *Central Dogma*, have been essential steps to understand information storage and transfer within and among cells and organisms. Furthermore, the current understanding of the infectivity of prion-proteins after misfolding is summarized succinctly. Finally, prion-like amyloids and functional amyloids, as found in yeast and bacteria, will be discussed.

## 1. Introduction

**Figure 1:**
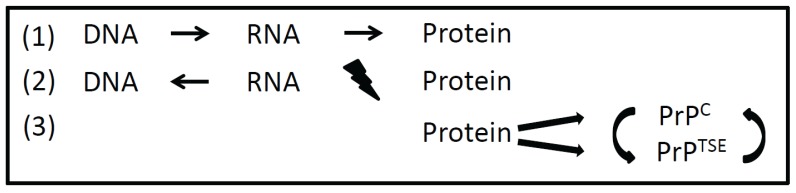
Information transfer between different macromolecules. According to the *Central Dogma* formulated by Watson and Crick, DNA in transcribed into RNA and then translated into protein (**1**). Reverse transcriptase also allows the transfer of information from RNA to DNA. An information transfer from protein back to nucleic acid is precluded (**2**). Prions transfer information in a self-replicating manner from protein to protein by a distinct misfolded protein conformation (**3**). PrP^C^, cellular prion protein; PrP^TSE^, misfolded isoform of the prion-protein (TSE = transmissible spongiform encephalopathy).

Prions (proteinaceous infectious particles, PrP^TSE^ (TSE = transmissible spongiform encephalopathy)) are the causative agents of fatal neurodegenerative diseases as bovine spongiform encephalopathy (BSE) in cattle, scrapie in sheep, chronic wasting disease (CWD) in cervids and Creutzfeldt-Jakob disease in humans [1,2]. In a process that can occur sporadically, by genetic mutations or by the uptake of prions, the cellular prion protein (PrP^C^) is structurally transferred in a misfolded (then pathogenic) isoform (PrP^TSE^) [3]. In a self-propagating process, more disease-associated, transmissible PrP^TSE^ becomes accumulated in the central nervous system causing progressive spongiform changes [4]. Prions are pathogens that fundamentally differ from bacteria, viruses or fungi as they are thought to consist essentially of host encoded prion protein lacking a coding nucleic acid. PrP^TSE^ is characterized by an increased β-sheet content and tends to form highly ordered amyloid structures [5]. Similarly to prion diseases, neurodegenerative diseases such as Alzheimer’s disease and Parkinson’s disease are neuropathologically characterized by the aggregation and deposition of misfolded endogenous proteins in the central nervous system. Albeit proteins from different neurodegenerative diseases differ in terms of amino acid sequences and native folds, their disease-associated proteins form extracellular amyloid deposits or intracellular amyloid-like inclusions. Principally, amyloids need not to be related to diseases but can also be beneficial as in bacteria (functional amyloids) or yeast (prion-like proteins) where they contribute to cell stability or act as inheritable elements [6,7]. Amyloids can be transferred from cell to cell or even from organism to organism. At least in case of prion diseases, amyloids can transfer disease by the transmission of specific, structurally altered, isoforms of cellular proteins. The discovery of functional amyloids, prion-like proteins and prions (including different strains as outlined below) revealed the necessity to expand the *Central Dogma* of transcription and subsequent translation being the dominant ways of information transfer in and between living cells and organisms (Figure 1).

## 2. Expanding the *Central Dogma*

When the double helix structure of DNA (deoxyribonucleic acid) had been discovered by F. Crick and J. Watson in 1953, a macromolecule as hereditary material was manifested [8]. At this time it was unclear how genetic information is encoded in DNA and how this information is transferred to proteins. Also the function of RNA (ribonucleic acid) in gene expression remained to be resolved. Eight years later Crick published the hypothesis that a transfer of information from nucleic acid to nucleic acid or from nucleic acid to protein may be possible [9]. This brilliant idea still holds true in what we call transcription and translation today. Finally, it was stated that “*DNA makes RNA makes protein*”. The discovery of RNA-viruses and reverse transcriptase revealed that the information transfer from DNA to RNA needs not to be unidirectional. But according to the *Central Dogma* it was proposed that “*once information has passed into protein it cannot get out again*” [8]. Information transfer from protein back to nucleic acid and from protein to protein was precluded. As a transfer of information from protein to protein was proposed by the prion-hypothesis, and functional amyloids and prion-like proteins (as described below) were discovered, the *Central Dogma* (even after minor revision by Crick in 1970 [9]) had to be expanded (Figure 1). Since S. Prusiner has been awarded the Nobel Price for the prion hypothesis in 1997, the idea that a misfolded isoform of a cellular protein can transfer information onto other cellular prion proteins in a self-replicating manner became more and more accepted [10,11].

## 3. Prions as Information Carriers within and among Cells and Organisms

Many biological processes are driven by changes in protein conformation but prions are unique pathogens as they transmit disease by a distinct self-replicating misfolded isoform of the cellular prion protein [12,13,14,15]. They display a pathological, β-sheet rich conformation that tends to aggregation. The cellular form of the prion protein displays a different secondary structure with a low β-sheet content. PrP^C^ is not infectious, soluble in mild detergents and sensitive to protease digestion. According to a misfolding process, designated as nucleation-dependent polymerization, oligomers of PrP^TSE^ act as nucleation-seeds that recruit PrP^C^ and incorporate it, after misfolding, into an amyloid-like aggregate-structure [13]. In the “nucleation-phase” misfolded conformations of host encoded prion proteins become stabilized by oligomerization and form “nuclei” [16]. During the “elongation-phase” protofilaments are formed by further accumulation of prion proteins to the PrP^TSE^-nuclei. Once larger fibrils are formed they tend to break into smaller units during the “fragmentation phase”. Further cycles of elongation and fragmentation finally result in an exponential increase of PrP^TSE^.

PrP^TSE^ does not only spread from cell to cell but also from individual to individual as it naturally occurs in scrapie and CWD [17,18,19]. Prions can exist as different strains similarly to bacteria or viruses. Although sharing the same amino acid sequence, prions from one host can adopt different conformations. A prion strain is characterized by its specific PrP^TSE^-conformation, the potential of infection and by PrP^TSE^ spreading and deposition in the brain and other prion-associated tissues [20]. A high resolution structure for PrP^TSE^ is not available and the conversion process is still not understood in detail [21,22]. Different strains also occur in Alzheimer’s and Parkinson’s disease-associated aggregated proteins [23,24]. Similarly to prion diseases, the pathology of these protein misfolding diseases can vary when different strains are present as the protein conformation has a direct impact on disease establishment and progression.

Several cofactors, such as polyanions (nucleic acids and proteoglycans) and lipids are discussed in the literature to be involved in the prion infectivity process [11]. These cofactors obviously play a fundamental role as catalysts and may stabilize oligomers. Recently, Simoneau *et al.* published data that indicate a specific role of short non-coding RNA-molecules in the generation of prions [25]. They showed that originally innocuous recombinant prion protein could be converted to a prion-like conformation in the presence of small RNA-molecules isolated from prion fibrils.

## 4. Prion-Like Amyloids and Functional Amyloids in Yeast and Bacteria

The understanding of prion biogenesis has profoundly been increased by the discovery of prion-like phenomena in yeast. In mammals, amyloids can be associated with a group of devastating neurodegenerative diseases, but yeast prions do not result in cell death [26,27]. Over the past few years, the number of yeast prions has rapidly grown [28]. Yeast prions can act as heritable proteinaceous elements and are propagated epigenetically. In *Saccharomyces cerevisiae* the normal cellular proteins Sup35 and URE2 can be converted to the self-propagating amyloids PSI^+^ and URE3, respectively. Sup35 acts as a translation termination factor, an ability that becomes impaired upon conversion to PSI^+^. URE2 is a nitrogen catabolite repressor once transformed into URE3 allowing growth on poor nitrogen sources. Other yeast prions are involved in the regulation of transcription, translation and in the biogenesis of ribosomes [28]. Even though amyloids in yeast do not cause severe disease as in mammals, they share several features with prions as they are transmissible and continuously replicate their structure and disseminate their self-replicating activity [29,30]. Not all amyloids are prions, but several amyloids in yeast act prion-like [31].

In *Escherichia coli*, the curli-protein, that assembles on the outer cell-membrane, is an example for functional amyloids [32,33]. Organisms such as *E. coli* have developed the ability to direct amyloid formation spatially and temporally. Such functional amyloids fulfill a variety of important physiological roles and are not toxic to the organism that produces them. Curli is the main protein component in biofilms of Gram-negative bacteria and stabilizes the biofilm-matrix, is important for cell-adhesion, cell-cell contact and plays a role in immune response [34]. The curli specific genes are found in two operons. The major and minor curli subunits, CsgA and CsgB, are encoded by the *csg*BAC operon. Once CsgA is secreted across the outer membrane as an unstructured soluble peptide, it is templated into an amyloid on the cell surface by CsgB [34]. In contrast to mammalian prions and yeast-amyloids, bacterial amyloids are neither infectious nor transmissible therefore belonging to the group of functional amyloids.

## 5. Conclusions

Proteins tend to aggregate under specific conditions. Protein aggregation can occur *in vivo* and *in vitro*, ordered and disordered [31]. Amyloids are insoluble fibrous protein aggregates that are examples of ordered aggregates. In contrast, aggregated proteins found *in vivo* in inclusion bodies are disordered. Amyloids are highly ordered β-sheet rich protein assemblies that have been found in a variety of functional or pathogenic contexts. Mammalian prions are characterized by their ability to be infectious and self-replicating [35]. In humans they are associated with neurodegenerative diseases. While in yeast similar phenomena have been shown (prion-like amyloids), these proteins do not cause disease or cell-death [26]. In bacteria functional amyloids have been discovered that do not act in a prion-like manner [36]. In bacteria the timing, localization and structure of amyloid fibers is determined by dedicated molecular control systems. Those control systems can be turned on and off depending on the benefit of the cell under specific environmental conditions. In case of prion diseases, the onset of protein conversion is sporadic, genetically based or induced by the uptake of a misfolded prion protein isoform. Once the misfolding process has begun it cannot be stopped and finally leads to death.

Prion-like amyloids and functional peptides also occur in humans and mammals. While they can act as inheritable elements in yeast, prion like-amyloids in humans are mainly recognized in the context of disease (e.g., Alzheimer’s diseases, Parkinson’s disease) [37]. Recently published data provide evidence for a prion-like transmission of Aβ pathology in Alzheimer’s disease, and for α-synuclein causing multiple system atrophy in humans with parkinsonism, respectively [38,39]. An example for a functional amyloid in humans is Pmel17 playing an important role in the biosynthesis of the pigment melanin [40,41].

Thus, besides DNA and RNA, proteins can transmit information from cell to cell or even from organism to organism by distinct structural protein foldings (Figure 1). Besides nucleic acids, prions and prion-like proteins represent additional molecules for information storage and transmission. Information stored in distinct conformations of prion molecules can convert PrP^C^ into Prp^TSE^ in the course of infection. This expands the *Central Dogma* as it demonstrates the possibility of information transfer from protein to protein. Future research will elucidate whether amyloids (in particular functional amyloids) are more common in life than it is assumed so far.
